# Efficacy and Safety of Limosilactobacillus reuteri DSM 17938 in Infantile Colic: A Narrative Review From Pathogenesis to Clinical Practice

**DOI:** 10.7759/cureus.107824

**Published:** 2026-04-27

**Authors:** Kimberly Carvajal Méndez, Ana Gabriela Álvarez Barrientos, Freddy Gutiérrez Rivera, Daniel Aragón Leiva, Dennis Ulate Fuentes

**Affiliations:** 1 Faculty of Medicine, Universidad de Costa Rica, San José, CRI; 2 Faculty of Medicine, Universidad de Iberoamerica (UNIBE), San José, CRI; 3 Faculty of Medicine, Universidad Latina, San José, CRI

**Keywords:** dsm 17938, dysbiosis, fgids, functional gastrointestinal disorders, gastrointestinal microbiome, infantile colic, limosilactobacillus reuteri, probiotics

## Abstract

Infantile colic is one of the most common functional gastrointestinal disorders, affecting approximately 20% of infants worldwide. Characterized by paroxysmal episodes of inconsolable crying and fussing without an obvious cause, the condition imposes a significant psychosocial burden on the family unit, acting as a primary trigger for maternal postpartum depression, parental exhaustion, and premature cessation of breastfeeding. Despite its prevalence, traditional management in primary care remains heterogeneous and often ineffective, leaving a substantial therapeutic gap. Emerging research has identified gut dysbiosis as a cornerstone of its pathogenesis, where an imbalance in early microbial colonization, characterized by reduced diversity and a higher prevalence of gas-producing coliforms, triggers intestinal inflammation and visceral hypersensitivity through the gut-brain axis.

This narrative review synthesizes the current clinical evidence regarding the efficacy and safety of *Limosilactobacillus reuteri* DSM 17938 in the management of infantile colic. This specific probiotic strain exerts its therapeutic effects through multifaceted mechanisms, including the production of the antimicrobial metabolite reuterin, the modulation of gastrointestinal motility via microvesicles, and the reduction of low-grade gut inflammation. Clinical evidence from large-scale meta-analyses confirms that *L. reuteri *DSM 17938 is significantly superior to placebo, particularly in breastfed infants, where it is twice as likely to achieve a 50% reduction in crying time within 14-21 days of treatment. However, its efficacy appears less predictable in formula-fed populations, potentially due to the absence of synergistic factors found in human milk.

Clinical management in primary care should prioritize a rigorous diagnostic approach to rule out organic causes through a detailed physical examination and the application of the Rome IV criteria. The cornerstone of non-pharmacological management remains empathetic parental reassurance and education to mitigate the risk of infant abuse and support family well-being. When probiotic intervention is indicated, *L. reuteri* DSM 17938 represents a robust, evidence-based first-line therapy for healthy, full-term infants. Regarding safety, this strain maintains an excellent profile supported by its generally recognized as safe (GRAS) status, with no significant adverse effects reported in healthy populations. Nevertheless, recent clinical scrutiny emphasizes the need for caution and the use of pharmaceutical-grade products in vulnerable groups. Ultimately, *L. reuteri* DSM 17938 addresses both the biological triggers of colic and the psychosocial stability of the family unit.

## Introduction and background

Infantile colic is one of the most common functional gastrointestinal disorders (FGIDs) in infancy [1]. Historically defined by Wessel's Rule of Threes, the clinical landscape has shifted toward the Rome IV criteria. These criteria describe colic as paroxysmal episodes of fussing or crying that start and stop without an obvious cause, lasting three or more hours daily, occurring three or more days per week for at least one week, in infants under five months of age with no evidence of failure to thrive or fever [2]. While the transition to Rome IV criteria marked a pivotal advancement and has reduced overdiagnosis and shortened the time-to-diagnosis, the condition remains a challenge in daily clinical practice due to its inherent subjectivity.

Affecting approximately 20% of infants worldwide, colic is a leading cause of pediatric consultations and imposes a substantial economic burden on healthcare systems [1,2]. Beyond its clinical prevalence, the condition exerts a profound psychosocial toll on the family unit, frequently acting as a primary trigger for maternal postpartum depression, parental exhaustion, and the premature cessation of breastfeeding. Crucially, inconsolable crying is a recognized risk factor for shaken baby syndrome, elevating infant colic from a transient functional issue to a critical public health concern [1].

The pathogenesis of infantile colic remains elusive, though a multifactorial model is currently supported. This model suggests an interaction between gastrointestinal triggers, such as gut immaturity, dysmotility, and dysbiosis, and behavioral factors like maternal anxiety, parent-infant interaction, and infant temperament. Because diagnosis relies on clinical criteria and the exclusion of underlying pathologies, a thorough evaluation is essential to rule out organic causes, including gastrointestinal disorders, infections, or neurological disorders [1-3].

Despite its prevalence, primary care management remains heterogeneous and often ineffective. Conventional pharmacological interventions have failed to consistently outperform placebos, while other treatment options like dietary modifications, complementary therapies, manual therapies, and parental behavioral interventions lack robust comparative evidence [2,4]. Consequently, there is growing interest in modulating the infant gut microbiota; specifically, the administration of probiotic strains has emerged as a promising strategy [3].

This narrative review seeks to synthesize the current clinical evidence regarding the efficacy and safety of *Limosilactobacillus reuteri *DSM 17938 in the management of infantile colic, from its underlying pathogenesis to its application in clinical practice. A literature search was conducted using the PubMed and Google Scholar databases, focusing on articles and official regulatory documents published between 2006 and 2026. The search strategy employed keywords such as "infantile colic", "probiotics", and "*Limosilactobacillus reuteri* DSM 17938". We prioritized high-level evidence, including meta-analyses, randomized controlled trials, and official safety notices from regulatory agencies (e.g., Food and Drug Administration (FDA)), to ensure the clinical recommendations are based on the most current and robust data.

## Review

Pathophysiology of infantile colic

At birth, the gastrointestinal microbiota is immature and highly individual, shaped by multiple factors, including the uterine environment, delivery mode (vaginal vs. cesarean), and early life exposures. Gut dysbiosis, an alteration in microbial community structure compared to its healthy counterpart, is now considered a cornerstone in the pathogenesis of infantile colic [3,5,6].

The Dysbiosis Hypothesis

This hypothesis suggests that an imbalance in early neonatal gut colonization plays a central role in the development of colic. Affected infants often exhibit reduced microbial diversity and a delay in the colonization of beneficial commensals such as *Bifidobacterium* and *Lactobacillus*. This deficit compromises the production of short-chain fatty acids (SCFAs), essential for intestinal barrier integrity and immune modulation [6,7].

Concurrently, research has identified a higher prevalence of gas-producing bacteria, particularly within the *Proteobacteria* phylum (e.g., *Escherichia coli*). These coliforms promote excessive fermentation, increasing hydrogen gas production and abdominal distention. This imbalance is also linked to local and systemic inflammation, evidenced by elevated fecal calprotectin and increased inflammatory markers such as interleukin (IL)-8 and monocyte chemoattractant protein-1 [6,8].

Furthermore, the bidirectional communication within the gut-brain axis serves as a critical pathway in the pathophysiology of infantile colic, involving a complex interplay of neural, endocrine, immune, and humoral signaling. The gut microbiota significantly influences both central and enteric nervous system functions; specifically, dysbiosis may alter nociception and pain perception, potentially triggering paroxysmal crying. While the central nervous system modulates gastrointestinal motility, secretion, and immune tone, the microbiota can independently synthesize or regulate key neurotransmitters such as serotonin (5-HT), dopamine, and gamma-aminobutyric acid (GABA). These neuroactive compounds, along with microbial metabolites and endotoxins, can enter systemic circulation or stimulate enterochromaffin cells to release serotonin into the lamina propria, increasing colonic concentrations and further sensitizing the gut-brain signaling loop [6,7,9].

Probiotics in clinical practice

Definitions and Quality Standards

The World Gastroenterology Organisation (WGO) defines probiotics as live microorganisms that, when administered in adequate amounts, confer a health benefit on the host [10]. To ensure efficacy, clinical-grade probiotics must maintain precise taxonomic identity; purity, ensuring manufacturing processes are free from pathogens; and potency, which guarantees that a sufficient number of colony-forming units (CFUs) remain viable until the end of the product's shelf-life. From a safety perspective, clinical-grade probiotics must demonstrate a lack of transmissible antibiotic resistance genes to prevent horizontal gene transfer within the host's gut microbiota. Finally, to exert a therapeutic effect, these microorganisms must demonstrate resilience against the harsh environment of the gastrointestinal tract, specifically the ability to withstand gastric acidity and survive exposure to bile salts to ensure successful colonization [11].

Strain Specificity and Nomenclature

The nomenclature and classification of probiotics have undergone significant revisions to reflect a more precise understanding of their genetic and metabolic diversity. Historically, many beneficial strains were grouped under the broad genus *Lactobacillus*; however, a major taxonomic reclassification in 2020 reorganized this group into 25 distinct genera (including 23 novel genera). Consequently, the species formerly known as *Lactobacillus reuteri* was officially renamed *Limosilactobacillus reuteri*. In clinical practice, it is imperative to distinguish between the genus (*Limosilactobacillus*), the species (*reuteri*), and the specific strain (DSM 17938), as therapeutic efficacy and safety profiles are strictly strain-specific [10,11,12].

The therapeutic effects of probiotics are categorized into widespread, species-specific, and strain-specific mechanisms. Widespread mechanisms, common to most probiotic strains, include the normalization of intestinal communities, competitive exclusion of pathogens, and regulation of intestinal transit. In contrast, specific mechanisms are unique to certain strains and responsible for distinct clinical outcomes. This fundamental principle precludes the extrapolation of clinical data between different strains, even within the same species; physiological effects demonstrated by a well-characterized strain cannot be assumed for generic counterparts, as minor genetic variations can result in vastly different metabolic activities. Consequently, major international guidelines from the WGO and the European Society for Paediatric Gastroenterology, Hepatology and Nutrition (ESPGHAN) emphasize that clinical recommendations for conditions such as infantile colic must be based on evidence derived from the exact strain studied, rather than at the species or genus level [10,13,14].

*Limosilactobacillus reuteri* DSM 17938: mechanism of action

Antimicrobial Properties and Reuterin Production

*Limosilactobacillus reuteri *is a key member of the lactic acid bacteria (LAB) group that naturally inhabits the gastrointestinal tract of humans and other animals. Originally isolated from human breast milk, *L. reuteri* DSM 17938 is a daughter strain of the parent strain ATCC 55730. To ensure clinical safety and prevent the horizontal transfer of antibiotic resistance, the original plasmids were removed to develop the DSM 17938 strain [15,16]. Its primary therapeutic feature is the synthesis of reuterin during glycerol fermentation. Reuterin is a broad-spectrum antimicrobial metabolite that inhibits Gram-positive and Gram-negative bacteria, as well as various enteropathogens. In colicky infants, this activity helps suppress gas-producing coliforms, thereby modulating the gut environment and reducing the microbial triggers associated with paroxysmal crying [15].

Modulation of Motility, Inflammation, and Pain

Beyond these antimicrobial properties, *L. reuteri DSM *17938 plays a significant role in regulating gastrointestinal motor function. Experimental data demonstrate that this strain can accelerate gastric emptying, reduce gastric distention, and decrease the frequency of regurgitation in infants. These therapeutic benefits are largely mediated by microvesicles (µV), membrane-derived structures containing proteins, phospholipids, and signaling molecules, which recapitulate the bacteria's effect on intestinal motility through inter-kingdom signaling. Specifically, *L. reuteri *DSM 17938 exerts region-specific effects, decreasing the frequency of contractions in the jejunum while increasing colonic motor activity. This modulation of the enteric nervous system (ENS) and contractile patterns suggests that the strain acts as a biological regulator of gut transit, directly addressing the delayed gastric emptying and dysmotility observed in colicky infants [16].

Furthermore, clinical evidence indicates that this strain effectively mitigates low-grade gut inflammation [8,17,18]. Systematic reviews and meta-analyses have demonstrated a correlation between probiotic treatment and a significant reduction in calprotectin concentrations, suggesting that this strain promotes the resolution of local inflammation and strengthens the intestinal barrier. By modulating microbial abundance and reducing the secretion of pro-inflammatory markers, *L. reuteri* DSM 17938 not only improves clinical symptoms but also addresses the underlying biological distress and compromised mucosal integrity associated with neonatal dysbiosis [6,18].

Finally, *L. reuteri *DSM 17938 acts as a biological modulator of the gut-brain axis. In infants, this strain can influence visceral nociception by interacting with the ENS. Specifically, certain probiotic metabolites can modulate the excitability of sensory neurons and the expression of pain-signaling receptors, such as the transient receptor potential vanilloid 1 (TRPV1). By increasing the threshold for visceral pain and regulating the release of neurotransmitters like 5-HT, *L. reuteri* DSM 17938 effectively reduces the distress and paroxysmal crying associated with gut distention and dysmotility [19].

Clinical efficacy of *L. reuteri *DSM 17938

The clinical superiority of *L. reuteri *DSM 17938 is underscored by robust statistical data from large-scale evidence syntheses. According to the individual patient data (IPD) meta-analysis by Sung et al., infants treated with this strain were 1.7 times more likely than the placebo group to achieve treatment success, defined as a reduction in daily crying or fussing time of at least 50%, by day 21 (95% CI: 1.4 to 2.2). On average, these infants experienced a significant reduction of 25.4 minutes of crying per day (95% CI: -47.3 to -3.5) [20]. This body of evidence has been further strengthened by a 2024 meta-analysis by Vaz et al., which reported that probiotics resulted in an average crying reduction of 51 minutes per day (p=0.001). Specifically, the administration of *L. reuteri* DSM 17938 demonstrated a significant reduction of 64.66 minutes (p=0.03) [21]. Furthermore, a network meta-analysis (NMA) by Gutiérrez-Castrellón et al. reported a significant weighted mean difference (WMD) in crying duration of -51.3 hours (95% CI: -72.2 to -30.5 hours; p<0.0001) compared to the control group by the end of the intervention [22]. While these results are compelling, the substantial magnitude of this effect must be interpreted with caution due to the inherent heterogeneity among the included studies, such as differing definitions of crying and variations in infant baseline characteristics. Nevertheless, this cumulative improvement over the treatment period highlights the strain's superior efficacy when compared to alternative pharmacological or dietary interventions. Collectively, these data confirm that the administration of *L. reuteri* DSM 17938 significantly reduces mean crying and fussing times in colicky infants compared to placebo groups and provides a predictable and statistically significant alleviation of symptoms within 14-21 days [14,23,24]. 

Efficacy by Feeding Type and Parental Impact

A critical finding in the literature is the disparity in efficacy based on the infant's diet. In the Sung et al. meta-analysis, the intervention effect was particularly dramatic in the breastfeeding subgroup, yielding a number needed to treat (NNT) of 2.6 (95% CI: 2.0 to 3.6) [20]. This is corroborated by Vaz et al., who identified a substantial reduction of 74.28 minutes of crying per day in exclusively breastfed infants (p=0.0003), compared to a more modest reduction of 48.04 minutes in those with mixed feeding (p<0.00001) [21]. Conversely, the effect in formula-fed infants has been more heterogeneous, with some trials demonstrating modest improvements while others failed to show a statistically significant superiority over placebo [10,14,17]. This discrepancy may be attributed to inherent differences in the gut microbiota of formula-fed infants, who often exhibit higher levels of *Clostridium *species compared to breastfed infants, potentially creating a less receptive environment for the colonization of *L. reuteri *[6,23]. Furthermore, breast milk contains natural human milk oligosaccharides and growth factors that act synergistically with the probiotic strain, elements that are often absent or present in different concentrations in standard infant formulas [14]. Consequently, while *L. reuteri* DSM 17938 remains a viable therapeutic option, current guidelines suggest its clinical impact may be less predictable in infants receiving exclusive formula feeding. Recent technical consensuses reinforce this stance, concluding that although certain strains show promising results, the available evidence is still insufficient to establish a formal recommendation in favor of the systematic supplementation of infant formulas with probiotics for the treatment of colic [25].

Beyond the quantitative reduction in crying duration, the administration of *L. reuteri *DSM 17938 exerts a profound impact on family dynamics and parental well-being. Excessive infant crying is a recognized trigger for maternal postpartum depression and, in extreme cases, infantile abuse. By providing a predictable clinical response, particularly in breastfed infants, this probiotic intervention helps restore household stability and may reduce the risk of premature breastfeeding cessation and severe behavioral disturbances. Therefore, the therapeutic value of *L. reuteri *DSM 17938 extends beyond gastrointestinal relief, serving as a critical support tool for the overall mental health of the family unit during the first months of life [1].

Safety and tolerability of *L. reuteri *DSM 17938

The safety of therapeutic interventions in the neonatal period is paramount. Most probiotic strains have been granted the generally recognized as safe (GRAS) status by the US FDA, reflecting a long history of safe use in healthy infants [11]. Specifically, the safety of *L. reuteri *DSM 17938 is supported by its inclusion in the FDA GRAS notice inventory (GRN no. 254 and no. 440), following independent evaluations that concluded the strain is safe for its intended use in infant formulas and various food products [26,27]. This safety profile is further contextualized by the WGO 2023 Global Guidelines, which emphasize that while these microorganisms are generally well-tolerated in healthy populations, strict caution is required in vulnerable groups [10].

For the management of infantile colic in healthy infants, where the WGO awards *L. reuteri* DSM 17938 its highest level of evidence (Level 1), clinical trials have demonstrated no significant adverse effects, provided that products meet rigorous quality standards for strain identity and viability [10,20,23,28]. Furthermore, the deliberate removal of antibiotic resistance-carrying plasmids during the development of the DSM 17938 strain ensures that there is no risk of horizontal gene transfer [11].

However, the scientific community has adopted a more cautious perspective following a landmark systematic review by Bafeta et al., which highlighted a widespread deficiency in the reporting of harms and adverse events in probiotic trials [28]. This clinical scrutiny was further intensified by a 2023 FDA safety alert regarding the risk of probiotic-associated sepsis in highly vulnerable populations, such as preterm neonates [29]. Therefore, while *L. reuteri* DSM 17938 remains a safe and well-tolerated intervention for the management of infantile colic in healthy infants, clinicians must prioritize pharmaceutical-grade products and maintain a cautious approach when treating infants with significant comorbidities or immunocompromised states [10].

Clinical management of infantile colic in primary care

Diagnostic Approach and Parental Support

The clinical management of infantile colic in primary care must begin with a rigorous diagnostic approach to exclude organic causes. A detailed clinical history and a thorough physical examination are essential to identify warning signs, such as severe vomiting, back arching, abdominal distention, or failure to thrive, which necessitate immediate investigation. Once organic pathologies are ruled out, the diagnosis of infantile colic should be confirmed using the Rome IV criteria, which provide a standardized framework for identifying this FGID based on paroxysmal episodes of crying and fussing in otherwise healthy infants [1,2].

The cornerstone of non-pharmacological management is parental reassurance and education. Clinicians must validate the significant psychosocial stress experienced by caregivers, explaining the benign and self-limiting nature of the condition, which typically resolves by the fourth or fifth month of life. Providing emotional support and practical coping strategies not only is therapeutic for the family unit but also serves as a critical preventive measure against shaken baby syndrome, a severe complication of parental exhaustion and inconsolable infant crying [1,25].

Clinical Application and Recommendations

When intervention is indicated, *L. reuteri* DSM 17938 is the first-line probiotic recommended, particularly for breastfed infants. The standardized dosage is 10^8^ CFU per day (typically administered as five drops daily). Caregivers must be advised that the therapeutic effect is not immediate, requiring continuous treatment for 14-21 days to achieve maximum clinical improvement. Regarding administration, drops may be given directly or mixed with breast milk or formula, provided the liquid is not hot, as high temperatures compromise bacterial viability [10,14,21,23].

Conversely, certain traditional interventions are no longer recommended due to a lack of evidence or safety concerns. Simethicone has consistently failed to demonstrate superiority over placebo in clinical trials and is generally discouraged. More critically, dicyclomine hydrochloride is strictly contraindicated due to the risk of severe adverse effects, including apnea and seizures. Additionally, dietary modifications, such as the use of extensively hydrolyzed or amino acid-based formulas, should not be implemented empirically; they should be reserved only for cases where a strong clinical suspicion of cow's milk protein allergy (CMPA) exists [1,2].

Finally, effective primary care involves established follow-up and referral criteria. While most cases of colic can be managed successfully by the pediatrician or family physician, a referral to a pediatric gastroenterologist is warranted if the infant exhibits any of the aforementioned red flags (e.g., severe vomiting, back arching, abdominal distention, or failure to thrive) and any other signs of further organic causes during the initial assessment or does not respond to initial evidence-based management [1].

Limitations and Future Perspectives

Despite the robust evidence supporting *L. reuteri* DSM 17938, this review acknowledges inherent limitations in the current literature, primarily the significant heterogeneity across clinical trials regarding infant populations, study designs, and varying definitions of crying. Furthermore, while its benefits in breastfed infants are well-established, there remains a critical need for more strain-specific randomized controlled trials to clarify its efficacy and clinical impact in formula-fed populations. Future research should prioritize investigating the long-term effects of early microbiota modulation on pediatric health outcomes and the potential synergistic effects of combining this strain with other functional ingredients like human milk oligosaccharides.

## Conclusions

Infantile colic remains a complex clinical challenge that extends beyond a transient gastrointestinal disturbance, significantly impacting parental mental health and family stability. Current evidence identifies gut dysbiosis as a primary driver of this condition, where an imbalance in early microbial colonization triggers intestinal inflammation, gas production, and visceral hypersensitivity through the gut-brain axis. Understanding these underlying biological mechanisms has shifted the therapeutic paradigm toward targeted microbiota modulation. *Limosilactobacillus reuteri *DSM 17938 stands out as the most extensively studied and evidence-based intervention, demonstrating a predictable reduction in crying duration and a high rate of treatment success, particularly in breastfed infants.

In primary care settings, the management of infantile colic should prioritize a rigorous diagnostic approach to exclude organic pathologies, followed by empathetic parental reassurance. While* L. reuteri *DSM 17938 is a safe and effective first-line therapy for healthy infants, its clinical efficacy is less predictable in formula-fed populations, suggesting that synergistic factors in breast milk play a crucial role in its success. Furthermore, clinicians must remain vigilant regarding product quality and safety in vulnerable populations, ensuring that pharmacological or probiotic interventions are always secondary to comprehensive caregiver support and education. Ultimately, a multi-faceted approach that addresses both the infant's biological distress and the family's psychosocial needs is essential for the effective management of this common disorder.
